# Vision impairment is a risk factor for malnutrition in older long-term care residents

**DOI:** 10.1007/s41999-025-01279-9

**Published:** 2025-11-17

**Authors:** Satu K Jyväkorpi, H. Soini, K. H. Pitkala, R. K. T. Saarela, S. Muurinen

**Affiliations:** 1 Department of General Practice and Primary Health Care, University of Helsinki, Helsinki, Finland; 2 National Nutritional Council, Helsinki, Finland; 3 Finnish Food Authority, Ruokavirasto, Finland; 4 Social Services, Health Care and Rescue Services Division, Oral Health Care, City of Helsinki, Finland

**Keywords:** Vision, Nutritional status, Older residents, Chewing problems, Oral health

## Abstract

**Purpose:**

To examine how vision is associated with nutritional status in long-term residents.

**Methods:**

Voluntary residents of 47 assisted-living facilities and 7 nursing homes, including 2513 permanent long-term care residents (age ≥ 65 years) were examined by trained nurses about various health-related aspects. Nutritional status was assessed using the Mini Nutritional Assessment long version and vision was estimated using a question from 15D-health-related quality of life questionnaire.

**Results:**

18% of the participants were characterised by having vision impairment. Vision impairment was independently associated with residents’ malnutrition (OR 1.62, 95% [1.22, 2.15], *p* < .001). Chewing problems (OR 2.78, 95% [2.18, 3.55], *p* < .001) and Clinical Dementia Rating memory item (OR 2.20, 95% [1.68, 2.87], *p* < .001) were also associated with malnutrition.

**Conclusion:**

Vision impairment was associated with malnutrition, problems with oral health and poorer cognition. Vision impairment should be addressed when managing nutrition in this vulnerable population.

## Introduction

Long-term care residents often have cognitive and physical impairments accompanied by many chronic diseases [1]. Malnutrition is common in long-term care residents, and it leads to poor quality of life and increases morbidity and mortality [2]. Vision impairment, poor cognitive status, polypharmacy, gastro-intestinal symptoms, poor oral health, and swallowing difficulties often lead to weight loss and malnutrition in long-term care residents [2–4]. Vision impairment and malnutrition are both associated with poor self-reported health and mobility, and they may increase the risk of mortality [3–5]. In a previous study in Helsinki long-term care facilities, vision impairment was associated with malnutrition, poor cognition and chewing problems [6]. In recent years, the population in long-term care in Finland has changed substantially. Now residents are in poorer condition, have more cognitive and physical impairments and thus need more care and assistance than previously. We repeated the previous study performed 10 years earlier with this new resident population in the same setting [6]. We were especially interested in finding out how vision impairment is related to malnutrition and its associative factors.

## Methods

Volunteer residents from a sample of 47 assisted-living settings and 7 nursing homes in Helsinki, Finland were recruited for this study in 2017/2018. In all the institutions, registered nurses oversaw the units and constant 24/7 assistance was available. Assisted-living facilities offer a more home-like environment than nursing homes. The resident population is similar in both institutions with respect to age, comorbidities and dementia [7]. The inclusion criteria for this present study were as follows: age ≥ 65 years, living permanently in a long-term care setting, sufficient demographic information available such as age, sex, nutritional status, comorbidities, vision, oral health and informed consent available. Nurses who collected the data and carried out nutritional assessment underwent training given by the study personnel. The participants’ weights were measured, whereas their heights and demographic information were obtained from the medical records. The body mass index (BMI) was calculated as weight divided by height squared (kg/m2). Charlson Comorbidity Index was calculated using diagnoses retrieved from medical records. The cognitive status of the residents was measured using the Clinical Dementia Rating (CDR) Memory item. For statistical purposes, the memory item was recoded as no dementia or mild dementia = 1, moderate or severe dementia = 2. Nutritional status was assessed using the Mini Nutritional Assessment (MNA) long version [4]. In addition, several questions associated with oral health were assessed. These included: (1) chewing problems (yes, no); (2) dry mouth (yes, no); (3) dysphagia (yes, no) and psychological well-being with Psychological Well-Being score [8]. Vision impairment was estimated using a question from 15D health-related quality of life (HRQoL) questionnaire; (1) I see normally, i.e. I can read newspapers and TV text without difficulty (with or without glasses). (2) I can read papers and/or TV text with slight difficulty (with or without glasses). (3) I can read papers and/or TV text with considerable difficulty (with or without glasses), (4) I cannot read papers or TV text either with glasses or without, but I can see enough to walk about without guidance. (5) I cannot see enough to walk about without a guide, i.e. I am almost or completely blind [10]. Options 4–5 were considered vision impairment and for statistical purposes the options were changed to 1 to 3 = adequate vision and 4 to 5 = vision impairment. In case of moderate to severe dementia, participants’ proxy most familiar with the older person’s condition and vision provided the response [9].

Ethics approvals were obtained from the Ethics Committee of the Department of Medicine at Helsinki University Hospital and the City of Helsinki (HUS/2042/2016). Informed written consent was asked from all participants, or in cases of moderate to severe dementia (MMSE < 20 points), from their closest proxies.

## Results

In total, 2513 residents, 64% of all long-term care residents in Helsinki, partook in the study. 18% of the participants were characterised of having vision impairment. Malnutrition according to MNA was more frequently encountered in those with vision impairment of whom 27% was classified as malnourished compared to 15% of those with normal vision (*p* < 0.001) (Table 1). In addition, those with vision impairment were older, had lower BMI and poorer cognition. Vision impairment was associated with chewing problems, dry mouth and dysphagia compared to persons with normal vision.

**Table 1:** Baseline characteristics of residents in long-term facilities divided according to their vision

Characteristics	Normal vision	Vision impairment	p—value
Participants, number (%)	2065 (82)	448 (17)	< 0.001^1^
Age, years	82.8 (8.6)	85.8 (8.8)	< 0.001
Sex, %			0.330
female	73	27	
male	75.2	24.8	
BMI, kg/m^2^, (SD)	25.3 (5.2)	23.6 (5.0)	< 0.001
CDR, memory, %			< 0.001
moderate or severe dementia	54.5	73.7	
MNA, score, mean (SD)	20.4 (3.6)	18.9 (3.6)	< 0.001
MNA (%)			
Normal nutritional status	20	11	< 0.001
Risk of malnutrition	65	61	
Malnourished	15	27	
Self-rated health, % good self-rated health	80.3	19.7	< 0.001
Charlson comorbidity index, (SD)	8.9 (3.6)	8.4 (3.7)	0.003
PWB, score, (SD)	0.72 (0.2)	0.68 (0.3)	0.003
Chewing prolems, %	26	45	< 0.001
Dry mouth, %	14	23	< 0.001
Dysphagia, %	19	30	< 0.001

^1^Statistical tests: Differences between groups were analyzed using the Chi2 -test or Fisher’s exact test for categorical variables and t-test for continuous variables.

BMI = body mass index; MNA = Mini Nutritional Assessment^4^; PWB = Psychological Well-Being.^8^

In logistic regression analysis controlling for age and gender, chewing problems, cognition and Charlson Comorbidity Index, vision impairment was independently associated with residents’ malnutrition (OR 1.62, 95% [1.22, 2.15], *p* < 0.001). Chewing problems (2.78, 95% [2.18, 3.55], *p* < 0.001) and CDR memory item (OR 2.20, 95% [1.68, 2.87], *p* < 0.001) were also associated with malnutrition. (Table 2.)

**Table 2:** Variables associated with residents’ nutritional status

Odd’s ratio for nutritional status^1^			
Model	Odd’s ratio	95%	p- value
Age	1.00	[0.99, 1.02]	0.965
Sex	0.846	[0.64, 1.13]	0.251
Vision impairment	1.62	[1.22, 2.25]	< 0.001
cognition CDR^2^ memory > 1	2.196	[1.68, 2.87]	< 0.001
Chewing problems	2.781	[2.18, 3.55]	< 0.001
Charlson comorbidity index	1.019	[0.96, 1.11]	0.562

^1^Odd’s ratio was analyzed using logistic regression analysis.

^2^CDR = Clinical Dementia Rating.

## Discussion

In our study, those with vision impairment were more frequently malnourished compared to those with normal vision. In addition, vision impairment was independently associated with malnutrition in older long-term care residents. Malnutrition was also associated with dry mouth, dysphagia and chewing problems and poorer cognition.

There are very few previous studies about vision and malnutrition in older long-term care settings. In a previous study conducted in the same setting 10 years prior to this study, vision impairment was similarly associated with malnutrition [6]. In a British study, home-dwelling older people with vision impairment consumed less energy and other nutrients than recommended for their age group and when compared with an age-matched control group [10]. Systematic review of 14 studies of all age groups concluded that visual impairment significantly affects nutritional status. The included studies showed that visually impaired people had an abnormal body mass index (BMI); a higher prevalence of obesity and malnutrition, where obesity was more common in the younger age group and malnutrition in older people [11].

Visual exposure to appetizing foods strongly activates brain regions involved in craving and reward, especially when a person is hungry [12]. Poor vision, in turn, can affect food intake by reducing the enjoyment of eating [12]. Seeing food also triggers physiological responses such as increasing appetite by rising hunger hormone ghrelin levels and, thus promoting food consumption [13]. This response may be diminished in individuals with impaired vision. In a small experimental study, participants who ate while blindfolded consumed 22% less food than when eating without a blindfold [14]. Interestingly, despite the reduced intake, their subjective feeling of fullness matched that reported after the larger, non-blindfolded meal [14]. In addition, visual impairment in long-term care settings can also affect food intake by simply making it more difficult to locate the served foods on the table or in the plate, particularly if food does not stand out from the plate. Nurses may not have enough time to assist everybody needing help with eating during mealtimes and thus the food intake may be reduced with the visually impaired. Visual impairment may also negatively impact oral health, as those with poor sight tend to have poorer oral hygiene compared to those with normal vision [15]. Poor oral health itself is a known risk factor for malnutrition as it affects the ability to eat different types of foods.

The strengths of our study include its relatively large sample of long-term residents. All the measurements were performed by trained nurses and all the questionnaires and measurements used were validated. Moreover, residents’ demographic information and diagnoses were retrieved from verified medical records, which increases the reliability of our findings. The study is, however, not without limitations, as the cross-sectional design of the study prevented us from drawing conclusions about temporal relationships. Vision impairment was estimated using just one question from the 15D HRQoL questionnaire [9]. Although the questionnaire is well-validated and can be answered by proxy in case of dementia, a proper diagnosis by a doctor would have given more reliable information on the matter. Another limitation of our study is due to the study populations’ high prevalence of dementia. In case the resident was not able to answer questions about her or his condition due to moderate to severe dementia, participants’ proxy most familiar with the resident’s condition provided the responses, when possible. However, the nurses were aware of the residents’ conditions and were thus able to give reliable answers concerning, for example, information about residents’ vision and oral health-related questions.

Our study shows that there is still place for improvement in managing nutrition among those with vision impairment in the long-term care population. Oral health problems and vision impairment go hand in hand as both increase the risk of malnutrition. In conclusion, those with vision impairment were more likely malnourished, although the prevalence of malnutrition and its risk were high in this population in general. Vision impairment should always be considered a risk factor for malnutrition. Therefore, the residents’ nutritional status should be closely monitored and assistance with oral hygiene and when necessary, eating assistance and nutrition care provided.

## Data Availability

The data is owned by the City of Helsinki and it will not be given to third parties upon a request.
